# Strengthening the transformative potential of gender mainstreaming in global health

**DOI:** 10.1016/j.eclinm.2021.100858

**Published:** 2021-04-14

**Authors:** Flavia Bustreo, Anna Giulia Ponchia, Cecilia Rocco, Rachael Hinton

**Affiliations:** a Fondation Botnar, and former Assistant Director General of Family, Women's and Children's Health at the World Health Organization, Switzerland; b London School of Economics, United Kingdom; c University of Trieste, Italy; d Independent consultant, Switzerland

Over the past three decades the United Nations (UN) has made important strides to promote gender mainstreaming as a strategic approach for achieving gender equality commitments through toolkits, manuals and training [1]. Despite these efforts, the envisioned transformative potential of gender mainstreaming has fallen short across the UN and other global institutions [1,2]. Covid-19 has also proved that these efforts are not enough.

Inadequate leadership and coordination for the implementation of gender mainstreaming policies, inconsistent support from member states, and a shift in focus to technocratic solutions in place of transformative ideals continue to hamper gender mainstreaming efforts [3]. International institutions also remain male-dominated in norms, attitudes and procedures. For example only 25% of women lead in global health, despite making up 70% of the global workforce [4]. Moreover, Covid-19 has also imposed an extra burden on female health and social care workers, however, women have not been represented equally in decision-making bodies for Covid-19 responses [4]. As of January 2021, only seven women made up the 21 member WHO Emergency Committee on Covid-19 [5]. At the national level females make up a minority of governments’ Covid-19 task forces [6].

Gender equality is still largely treated as a separate issue or delegated to specialized agencies or divisions such as UN Women, the Division for the Advancement of Women, and the Inter-Agency Network on Women and Gender Equality. In response there have been calls for UN institutions to ‘do gender better’ [1].

Within global health, the World Health Organization (WHO) committed to a new strategy to monitor its performance and accountability measures, strengthen engagement with civil society, and build capacity among senior and technical staff to support gender mainstreaming [7]. The WHO department of Gender, Equity and Rights was established to build programmatic and organizational mainstreaming capacity to address the impact of power relations and gender norms on people's health across the life course [7]. The focus is on mainstreaming gender as a relational concept that intersects with other drivers of inequalities, such as poverty, age, sexuality, ethnicity, and disability to impact on people's exposure to risk factors, vulnerabilities, and health outcomes [7].

In another example, the Pan American Health Organization, the WHO Regional Office for the Americas, adopted a policy in 2017 on Ethnicity and Health to address health inequalities among Indigenous peoples [8]. Indigenous women in particular have especially high rates of maternal mortality which are partly due to culturally inappropriate health services, stigmatization and discrimination due to gender and ethnicity [7]. The policy places emphasis on addressing the connection between ethnicity, gender, equity, and human rights in response to health service barriers rooted in discrimination, racism and exclusion [8]. Such integrated approaches to gender mainstreaming enable the development of a strong social justice agenda - to not only improve health and equity outcomes, but to also advance women's rights and empowerment [3,9].

The interconnected nature of the Sustainable Development Goals (SDGs) offers UN institutions a significant opportunity to support a transformative approach to gender equality. Covid-19 is also advocated as an opportunity to build a fairer society. However, challenges remain. Mainstreaming gender within global health institutions must not be seen as a technocratic exercise or the sole responsibility of a gender focal point. We support calls to increase institutional commitment at senior management levels for addressing gender equality and to strengthen accountability mechanisms for programming and results [1]. Robust measures for ‘doing gender better’ backed by resources and action are needed. Stronger working relationships with and across government and civil society will enable joint cooperation for gender mainstreaming within country strategies and plans.

To strengthen the transformative potential of gender mainstreaming, a feminist economic approach would help global health institutions address the intersection between power dynamics and the social, economic and political determinants of health delivery, resourcing, and decision-making for health [10]. Within the health workforce such an approach would bring attention to equal opportunities for women – pay, burden of work, career development – and the intersecting systems of discrimination that lead to inequalities faced by female health workers.

Gender equality must be pursued as a transformational agenda in itself (SDG5), as well as a means for achieving other goals, including health, economic growth, and education. This requires a strong conviction and collective momentum among global institutions and country partners to implement innovative mainstreaming strategies to tackle the structural causes of gender inequalities. We encourage moving beyond traditional gender training models to give space for new ideas and younger and diverse voices to address intersecting inequalities such as ethnicity, class, sexual orientation, and gender identity, including harmful male masculinity.

We are hopeful that the commitments from countries and other partners to the UN Generation Equality Forum result in advancements in equality for women and girls, because real transformative social change still remains to be seen.

## Declaration of Competing Interest

The authors have no conflicts to declare.
